# Experimental Inoculation of Coral Recruits With Marine Bacteria Indicates Scope for Microbiome Manipulation in *Acropora tenuis* and *Platygyra daedalea*

**DOI:** 10.3389/fmicb.2019.01702

**Published:** 2019-07-24

**Authors:** Katarina Damjanovic, Madeleine J. H. van Oppen, Patricia Menéndez, Linda L. Blackall

**Affiliations:** ^1^School of BioSciences, The University of Melbourne, Parkville, VIC, Australia; ^2^Australian Institute of Marine Science, Townsville, QLD, Australia; ^3^School of Mathematics and Physics, University of Queensland, Saint Lucia, QLD, Australia

**Keywords:** coral microbiome, probiotics, bacterial manipulation, assisted evolution, *Acropora tenuis*, *Platygyra daedalea*, host-symbiont specificity

## Abstract

Coral-associated microorganisms are essential for maintaining the health of the coral holobiont by participating in nutrient cycling and protecting the coral host from pathogens. Under stressful conditions, disruption of the coral prokaryotic microbiome is linked to increased susceptibility to diseases and mortality. Inoculation of corals with beneficial microbes could confer enhanced stress tolerance to the host and may be a powerful tool to help corals thrive under challenging environmental conditions. Here, we explored the feasibility of coral early life stage microbiome manipulation by repeatedly inoculating coral recruits with a bacterial cocktail generated in the laboratory. Co-culturing the two species *Acropora tenuis* and *Platygyra daedalea* allowed us to simultaneously investigate the effect of host factors on the coral microbiome. Inoculation cocktails were regularly prepared from freshly grown pure bacterial cultures, which were hence assumed viable, and characterized via the optical density measurement of each individual strain put in suspension. Coral early recruits were inoculated seven times over 3 weeks and sampled once 36 h following the last inoculation event. At this time point, the cumulative inoculations with the bacterial cocktails had a strong effect on the bacterial community composition in recruits of both coral species. While the location of bacterial cells within the coral hosts was not assessed, metabarcoding using the 16S rRNA gene revealed that two and six of the seven bacterial strains administered through the cocktails were significantly enriched in inoculated recruits of *A. tenuis* and *P. daedalea*, respectively, compared to control recruits. Despite being reared in the same environment, *A. tenuis* and *P. daedalea* established significantly different bacterial communities, both in terms of taxonomic composition and diversity measurements. These findings indicate that coral host factors as well as the environmental bacterial pool play a role in shaping coral-associated bacterial community composition. Host factors may include microbe transmission mode (horizontal versus maternal) and host specificity. While the long-term stability of taxa included in the bacterial inocula as members of the host-associated microbiome remains to be evaluated, our results provide support for the feasibility of coral microbiome manipulation, at least in a laboratory setting.

## Introduction

Scleractinian corals are responsible for building the three-dimensional structure of coral reefs through the deposition of their calcium carbonate skeletons, thereby providing habitat for over a quarter of all marine species (Spadling et al., 2001). Most scleractinian corals are colonial organisms, comprised of large numbers of interconnected polyps. Having persisted for over 200 million years (Veron, 1995), scleractinian corals owe this success to their symbiosis with photosynthetic microalgae in the family Symbiodiniaceae, which supply the host with most of their energy (Muscatine and Porter, 1977). In addition to these algal endosymbionts, corals form associations with a huge diversity of other microorganisms including bacteria, archaea, fungi and viruses (reviewed in Blackall et al., 2015). Altogether, the coral animal and associated microorganisms constitute a functional entity (analogous to a small-scale ecosystem; Pita et al., 2018) called the holobiont (Rohwer et al., 2002). Prokaryotes occupy various niches within the host, including intra- and extracellular spaces in tissues (Work and Aeby, 2014), the surface mucus layer, the gastric cavity and the skeleton (Sweet et al., 2010; Ainsworth et al., 2015). These communities exert numerous beneficial functions that are essential for the well-being of the coral animal, such as carbon, nitrogen, sulfur, and phosphorus cycling (reviewed in Bourne et al., 2016). Bacteria also protect corals from pathogens by occupying entry niches and secreting antimicrobial peptides (Bourne et al., 2016). Moreover, evidence from reciprocal transplantation experiments followed by short-term heat stress suggests that coral-associated bacterial communities are linked to intraspecific variation in coral heat tolerance (Ziegler et al., 2017). Maintaining an appropriate microbial community composition is undoubtedly key to preserve coral health, as detrimental alterations are usually observed to take place in diseased states and in response to adverse environmental conditions (Jones et al., 2004; Bourne et al., 2008; Littman et al., 2011; Tout et al., 2015).

Corals are suffering massive declines due to the global impacts of climate change and other anthropogenic disturbances (Hoegh-Guldberg, 2011; De’ath et al., 2012). Rising seawater temperature is a major cause of coral bleaching (the breakdown of the critical symbiosis between the coral host and its algal endosymbionts) and often leads to extensive coral mortality. The most severe bleaching on record for the Great Barrier Reef (GBR) occurred during the El Niño event of 2016, when ∼30% of coral present was lost (Normile, 2016; Hughes et al., 2017, 2018). Another mass bleaching event occurred the following year during a summer heat wave not related to El Niño (Hughes and Kerry, 2017), resulting in the loss of an additional ∼20% of coral cover (Ward, 2018). Climate models predict that most coral reefs in the world will experience similar extreme bleaching annually by the end of the century (van Hooidonk et al., 2016). Given their great ecological and cultural importance (Harrison and Booth, 2007; Blackall et al., 2015) and high economical value (Burke et al., 2011), the loss of reef-forming scleractinian corals would have severe consequences for coral reef ecosystems as well as for the coastal human populations depending on coral reefs.

While it is urgent to address the root causes of climate change, it is also essential to explore the possibility of augmenting coral tolerance and resistance to stress. Since environmental degradation may be occurring too fast for corals to adapt through natural selection (Hoegh-Guldberg, 2004; Hoegh-Guldberg et al., 2007), the concept of assisted evolution (AE) (Jones and Monaco, 2009) has been proposed as a strategy for coral reef conservation (van Oppen et al., 2015). AE aims to accelerate the rate of naturally occurring evolutionary processes, in order to develop corals better able to cope with current climate change trajectories (van Oppen et al., 2015). AE encompasses the manipulation of coral-associated symbionts, including members of the Symbiodiniaceae (Chakravarti et al., 2017) and prokaryotes (Damjanovic et al., 2017; Peixoto et al., 2017; Webster and Reusch, 2017; Epstein et al., 2019b).

Microbial inoculations have already been used in plants, humans, and a diversity of other host organisms. For example, plant growth promoting rhizobacteria (PGPR) are natural symbionts that colonize the rhizosphere, stimulate plant growth and development, and protect against biotic and abiotic stresses (reviewed in Singh and Singh, 2013; Gouda et al., 2018). In humans, probiotics are used both as supplementation to improve physiological functions in healthy individuals (Khalesi et al., 2018) and administered as treatments to patients suffering from various gastrointestinal disorders (Ringel et al., 2012). Rumen transfaunation (i.e., transfer of microorganisms from a healthy to a sick ruminant) is commonly conducted to enhance productivity and treat gastrointestinal dysbiosis in livestock (DePeters and George, 2014). Despite challenges inherent to employing microbial inocula in aquatic systems, such as establishing a suitable administration method, the potential of probiotics in aquaculture has been explored for over two decades (Gatesoupe, 1999; Verschuere et al., 2000). Probiotics are now widely used in this industry to promote animal growth, control disease, ameliorate water quality or augment stress tolerance (reviewed in Martinez Cruz et al., 2012; Hai, 2015). Microbiome manipulation in wildlife conservation is currently limited but recent applications offer hope for the protection of endangered species. For example, the amphibian cutaneous bacterium *Janthinobacterium lividum* secretes an antifungal metabolite effective against chytrid fungi which cause chytridiomycosis, a disease that has already decimated many frog populations worldwide (Rebollar et al., 2016). Bioaugmentation of antifungal bacteria through the inoculation of amphibian hosts holds promise to prevent the extinction of vulnerable populations. Similar to plants and other animals, exposing corals to certain microbial communities may trigger a beneficial shift in the symbiosis and render the holobiont more resilient to external pressures.

Although the notion of exogenously adding microbes to corals in a probiotic approach was suggested in 2009 (Teplitski and Ritchie, 2009), the field is still in its infancy but some early promising results have been obtained. Fragments of the coral *Mussismilia* *hartii* were inoculated with a bacterial consortium able to degrade water-soluble oil fractions, which reduced the negative impact of a simulated oil spill on the experimental corals (dos Santos et al., 2015). In another laboratory experiment, bacterial strains were selected for putatively beneficial traits including nutrient cycling, antioxidative capacities, and antagonistic activities against pathogens (Rosado et al., 2018). Inoculation of *Pocillopora damicornis* nubbins with the resulting consortium was able to partially mitigate coral bleaching and alleviate pathogenic infection (Rosado et al., 2018). Thus, coral bacterial community composition seems to be flexible to some extent and adjustable to benefit the host. Finally, a single exposure of coral larvae to the mucus-associated microbes of four different coral species resulted in divergent prokaryotic communities after 4 months of rearing in filter-sterilized seawater (Damjanovic et al., 2017). Even though the initial inoculum composition was not characterized, this experiment showed that coral-associated microbiomes could be influenced to develop in distinct directions following microbial dosing. Early coral life stages may be particularly suitable for targeted microbial inoculation, as the microbial composition and cell density in the surrounding environment strongly influences the microbiome acquired by juvenile corals (Apprill et al., 2009; Damjanovic et al., 2017). Further, the bacterial communities associated with early coral life stages tend to be more dynamic as compared to the associations harbored by adults (Littman et al., 2009a; Lema et al., 2014; Epstein et al., 2019a).

The objective of the present study was firstly to gain more insights into how associations between young corals and bacteria can be manipulated by targeted bacterial inoculations, and secondly, to examine whether different hosts are a determinant of the microbial community development. Coral recruits of two taxonomically divergent species, *Acropora tenuis* and *Platygyra daedalea*, were co-cultured in the same aquaria and simultaneously exposed to a cocktail composed of pure bacterial cultures. Metabarcoding of the 16S rRNA gene was used to assess the bacterial communities of the coral recruits following repeated inoculations. While undefined host factors were shown to play a role in the composition of coral recruit-associated bacterial communities, inoculated corals of both species were significantly enriched for some of the taxa that comprised the inoculum compared to non-inoculated control corals.

## Materials and Methods

### Coral Spawning and Rearing

Colonies of the corals *A. tenuis* and *P. daedalea* were collected off Falcon Island (S -18°46 E 146°32), Australia, and transported to the Australian Institute of Marine Science (AIMS) on the 4th of November 2017. The corals were deployed in 3000 L tanks containing running 0.4 μm filtered seawater (FSW). On the night of spawning (8th and 9th of November 2017 for *A. tenuis* and *P. daedalea* respectively), setting colonies were isolated in 80 L plastic bins filled with 0.4 μm FSW. Once released, gamete bundles were cautiously scooped from the water surface and washed over 60 μm plankton mesh to separate eggs from sperm. Gametes from six *A. tenuis* and 12 *P. daedalea* colonies were mixed separately for each coral species and left to fertilize for 2 h. The embryos were washed again with FSW to remove sperm and transferred to replicate cylindrical 60 L larval rearing tanks, which contained flow-through 0.1 μm FSW at 28°C and a low level of aeration.

**FIGURE 1:**
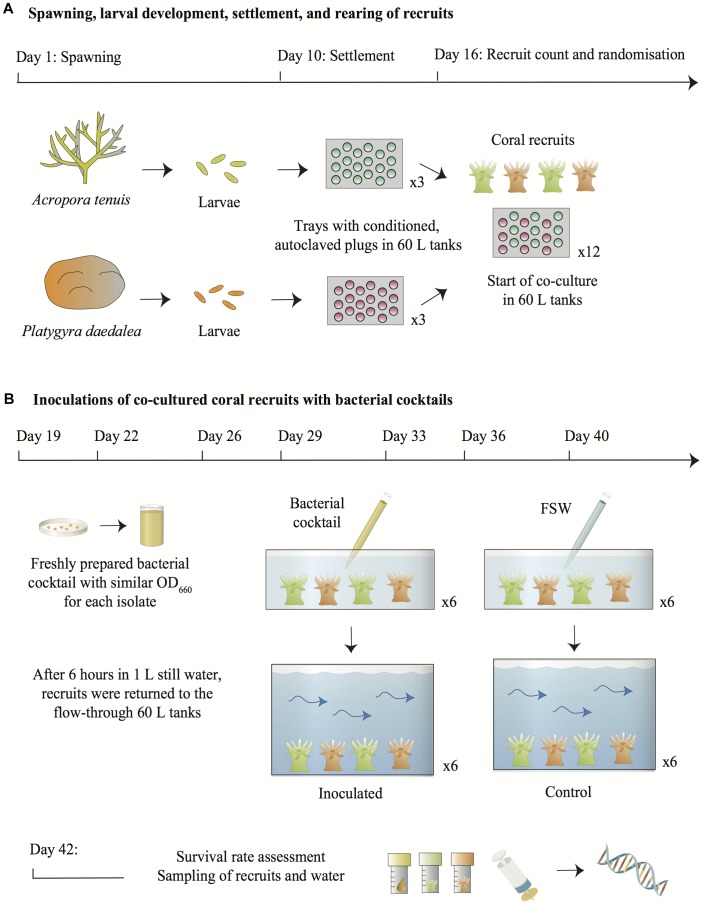
Overview of the experimental design. **(A)** Larvae from *A. tenuis* and *P. daedalea* were independently settled on plugs pre-conditioned with a microbial biofilm and CCA and subsequently sterilized. Six days post-settlement, recruits were counted and randomized across twelve 60 L tanks containing flow-through FSW. **(B)** On each inoculation day, bacterial pure cultures grown on MA from the frozen stocks were suspended in FSW and adjusted to similar densities in order to prepare a fresh bacterial cocktail. Recruits from each 60 L tank were placed in containers filled with 1 L of static FSW. The bacterial cocktail was added to six containers using a serological pipette to inject 10 mL of the suspension into the water directly above the recruits. The bacterial density in the inoculated containers was estimated at 10^5^–10^6^ cells/mL. Recruits from the control treatments were handled in the same manner, except that FSW was added to the containers instead of the bacterial cocktail. After 6 h in static water, recruits were transferred back into the 60 L flow-through tanks. Two days following the last inoculation, all recruits were counted, sampled from the 60 L tanks together with 1 L of surrounding water and snap-frozen for DNA extraction and 16S rRNA gene metabarcoding. Symbols for diagrams were modified from the Courtesy of Integration and Application Network (2018).

Nine days post spawning, approximately 9000 larvae from each species were distributed across three 60 L tanks (i.e., three settlement tanks for *A. tenuis* larvae and three different settlement tanks for *P. daedalea* larvae) containing flow-through 0.4 μm FSW at 27.5°C. The tanks were set under a 12 h light/dark illumination cycle reaching a maximum light intensity of 70 μmol⋅m^-2^⋅s^-1^ after 5 h ramping. Aragonite settlement plugs conditioned with a microbial biofilm and crustose coralline algae (CCA) and subsequently autoclaved had been placed in PVC trays on the bottom of the tanks. Six days after *A. tenuis* and *P. daedalea* juveniles were settled on the plugs in their respective tanks, the total number of recruits was counted under a dissecting microscope and plugs were randomized into 12 PVC trays. From this point onwards, *A. tenuis* and *P. daedalea* recruits were therefore co-reared on the same trays. The latter were spread across 12 new experimental 60 L tanks, set under the same light and temperature conditions, with a flow rate of 1 L/min (i.e., each hour the water volume completely changed). Water did not recirculate between these 12 tanks, as each one of them had different inflow and outlet pipes. In total, an average of 80 *A. tenuis* recruits and 47 *P. daedalea* recruits per replicate tank were available at the start of the inoculation experiment (Figure 1A). All recruits were tracked and counted again at the end of the experiment to assess survival rate.

### Preparation of Bacterial Pure Cultures

The inoculation cocktail was made from pure cultures of bacteria belonging to the genera *Acinetobacter*, *Bacterioplanes*, *Marinobacter*, *Paracoccus*, *Pseudoalteromonas*, *Pseudovibrio*, and *Vibrio*, which had been previously isolated from marine invertebrates and stored as frozen stocks (Table 1). As the goal of the present study consisted in assessing the potential of manipulating the microbiome of juvenile corals, the bacteria used were not selected for putative beneficial properties. Rather, we aimed to test to what extent the bacteria harbored by young recruits could be influenced by targeted inoculation as further support for the proof-of-concept of coral microbiome manipulation. The use of isolates from various marine organisms provides information about a range of bacterial sources that could be taken up by given coral species and hence how flexible these symbiotic partnerships are.

**Table 1:** Characteristics of the bacterial strains used to generate the inoculation consortium.

Bacterial identity	Closest relative	% identity from BLAST of X nt of the 16S rRNA gene	GenBank accession	Isolation source
*Acinetobacter sp.*	*A. baumannii*	99.9% from 1466 nt	MH744724	Healthy coral, GBR, Australia
Oceanospirillales isolate	*Bacterioplanes sanyensis*	99.7% from 1410 nt	MH744725	Healthy coral, GBR, Australia
*Marinobacter sp.*	*M. sediminum*	98.6% from 1512 nt	MK088251	Laboratory-bred anemone *Exaiptasia pallida*, Australia
*Paracoccus marcusii*	(Identified based on whole genome data)	–	MH744726	Healthy coral, GBR, Australia
*Pseudoalteromonas sp.*	*P. shioyasakiensis*	99.8% from 1403 nt	MK088250	Laboratory-bred anemone *Exaiptasia pallida*, Australia
*Pseudovibrio denitrificans*	(Identified based on whole genome data)	–	KX198136	Healthy coral, GBR, Australia
*Vibrio harveyi*	This is the type strain: DSM No 1963	–	X56578^∗^	Dead luminescing amphipod *Talorchestria* sp., United States


*The references for these strains are specified in the main text. nt, nucleotides. ^∗^GenBank accession number for the whole genome shotgun sequence: BAOD01000001.*

Pure cultures of *Marinobacter* sp. and *Pseudoalteromonas* sp. were isolated from the anemone *Exaiptasia pallida* maintained at the University of Melbourne (Table 1). Single anemones were transferred from the maintenance culture dish into 0.2 μm-filtered Red Sea Salt water (Red Sea^TM^) (FRSSW) and left static for 30 min in the dark to rinse external seawater. Each anemone was then gently transferred to a sterile glass homogenizer and homogenized with 1 mL FRSSW. Serial dilutions from 10^-1^ to 10^-4^ of the anemone homogenates in FRSSW were prepared and 50 μL were spread onto replicate plates of marine agar (MA - Difco^TM^ Marine Agar 2216). MA plates were incubated in the dark at 26°C for 1 week when individual, clearly separated colonies were 16-streak inoculated to new MA plates and incubated in the dark at 26°C for 2–3 days. This process was repeated until pure cultures were obtained, which were preserved at -80°C on cryobeads (Protect Microorganism Preservation System, Thermofisher, Cat# TS/80-MX).

Pure cultures of *Acinetobacter* sp., an Oceanospirillales isolate (used in van de Water et al., 2018), *Paracoccus marcusii* and *Pseudovibrio* *denitrificans* (Raina et al., 2016) were obtained from healthy coral colonies of *A. millepora* and *P. damicornis* (Table 1). The corals were collected from Pelorus Island and Davies Reef respectively (Great Barrier Reef: 18°33′S; 146°29′E and 18°51′S; 147°41′E), and maintained in aquaria at AIMS. Coral fragments (approximately 30 mm in length) were collected from each colony and washed in sterile artificial seawater (ASW) to remove loosely attached microbes. Coral tissue/microbe slurries were produced by airbrushing (550 kPa) each coral fragment into 5 mL of ASW and a dilution series was spread plate inoculated immediately onto modified minimal marine agar (1% bacteriological agar, 25 g of NaCl, 0.7 g of KCl, 0.05 g of KH_2_PO4, 1 g of NH_4_NO_3_, 1 g of MgSO_4_⋅7H_2_O, 0.2 g of MgCl_2_⋅H_2_O, 0.02 g of CaCl_2_⋅2H_2_O, 0.005 g of FeEDTA, 1 g of Tris, 5 g of sodium succinate, 1.35 g of glucose in 1 L of distilled water). After 2 days of incubation at 28°C, single bacterial colonies were transferred into Difco Marine Broth (BD^TM^, United States) and grown overnight in a shaking incubator at 28°C. Liquid cultures were re-streaked onto minimal marine agar; the procedure was repeated until pure cultures were obtained, which were stored at -80°C in 20% glycerol.

*Vibrio harveyi* was purchased from the Leibniz Institute DSMZ – German Collection of Microorganisms and Cell Cultures (Table 1).

### Inoculation of Coral Recruits With Bacteria and Sampling

Bacterial inoculation of the coral recruits was repeated seven times at regular intervals between November 27th and December 18th 2017 (Figure 1B). A fresh bacterial cocktail was prepared from the pure cultures before each inoculation event. Approximately 72 h before each inoculation, the seven bacterial cultures were revived from frozen stocks, streaked onto marine agar (Difco^TM^ Marine Agar 2216, BD) and incubated at 28°C. On the morning of coral recruit inoculation, sterile inoculation loops were used to collect bacterial colonies from the agar plates and cells of each strain were suspended separately in 9 mL 0.22 μm FSW. Optical density (OD_660_) of the suspensions was measured with a NanoDrop1000 spectrophotometer and cellular density was estimated using the calculation reported in Agilent Genomics (2018). Therefore, a cell density-OD_660_ value standard was used to infer cellular density. The seven bacterial suspensions were adjusted to 10^7^–10^8^ cells/mL (as estimated from the measured OD_660_: 0.082–0.21), either by dilution with additional 0.22 μm FSW or by introducing more cells in the suspension using a sterile inoculation loop. Nine mL of each suspension were then combined in a 100 mL sterile glass bottle. The optical density of the final bacterial cocktail was measured again to verify the collective cellular density. While optical density measurements do not distinguish between viable and dead bacterial cells, we assumed that the bacteria used to prepare the inoculation cocktails were alive as they were collected from freshly grown cultures. All OD_660_ measurements are summarized in Supplementary File 2.

At 11:00 am on the inoculation days, all trays with recruits on CCA plugs from each of the twelve 60 L tanks were transferred into twelve 3 L plastic containers filled with 1 L 0.4 μm FSW and maintained at 27.5°C. Ten mL of the bacterial cocktail were inoculated to six of the 3 L containers by pipetting the suspension directly into the 1 L of water above the recruits. The final bacterial density in the inoculated containers was 10^5^–10^6^ cells/mL (corresponding to a 1:100 dilution of 10^7^–10^8^ cells/mL present in the cocktail). To each of the remaining six 3 L containers, 10 mL of FSW were pipetted into the water and thus served as controls. All recruits were left in the static 3 L containers for 6 h before being returned to the 60 L flow-through tanks. The trays were handled manually wearing different pairs of ethanol-sterilized gloves between each replicate. On the first day of bacterial inoculation, recruits from all tanks were also exposed to a pure culture of Symbiodiniaceae *Cladocopium goreaui* (formerly known as type C1; LaJeunesse et al., 2018), strain SCF055-01.10 (GenBank accession number MK027323) at a density of ∼8,500 cells/mL. The *C. goreaui* pure culture was obtained from the AIMS Symbiont Culture Facility. The algal cells had been grown in Corning cell culture flasks at 27°C using a photoperiod of 14 h: 10 h light to dark illumination cycle and 60 μmol⋅m^-2^⋅s^-1^. Cells were maintained in 0.2 μm FSW and Daigo’s IMK sterile culture medium for marine microalgae (Nihon Pharmaceutical Co., Ltd.). This Symbiodiniaceae species was chosen because it commonly associates with *A. tenuis* on the GBR (LaJeunesse et al., 2004; van Oppen et al., 2005). While *P. daedalea* more often hosts a different species of Symbiodiniaceae in the wild (formerly known as type C3) (Fisher et al., 2011), it also naturally associates with *C. goreaui* (Abrego et al., 2009).

Thirty-six hours after the recruits had been placed back in the flow-through 60 L tanks following the last bacterial inoculation (at Day 40), recruits were counted under the dissecting microscope to assess survival rate and were also sampled for bacterial and Symbiodiniaceae community composition analysis. The microbial communities detected on the coral recruits therefore accounted for the cumulative effect of all seven inoculations. Degraded cells or residual DNA from the inocula surrounding the recruits were minimized in the samples, because the water flow of 1 L/min replaced the entire volume of the 60 L tanks 36 times. *A. tenuis* and *P. daedalea* recruits from each tray were removed from the plugs with a sterile scalpel blade, rinsed with 0.22 μm FSW and placed into cryovials for snap-freezing with liquid nitrogen. Bacterial and Symbiodiniaceae communities were also assessed in 1 L of water collected from each recruit maintenance tank and filtered through a 0.22 μm Sterivex^TM^ filter using a peristaltic pump. Samples of the seven bacterial inocula and the *C. goreaui* pure culture were collected as positive controls. The 16S rRNA genes and the nuclear DNA ribosomal internal transcribed spacer 2 (ITS2) were targeted in the metabarcoding analyses.

### DNA Extraction and Amplification

DNA was extracted from the samples following modifications of the protocol reported in Wilson et al. (2002). To provide enough prokaryote biomass for downstream PCR amplification of the 16S rRNA gene, ∼30 polyps for each recruit replicate sample were pooled for DNA extraction (i.e., both treatments provided six replicates of ∼30 polyps each), while 60 μL were used for the *C. goreaui* culture and each bacterial inoculum. Samples were placed in 1.5 mL sterile microcentrifuge tubes containing 250 μL of extraction buffer (100 mM Tris pH 9.0, 100 mM EDTA, 1% SDS, 100 mM NaCl). Ten μL of lysozyme at 10 mg/mL were added to all tubes and tubes were incubated at 37°C for 30 min. About 30 mg of sterile acid-washed glass beads (size 710 – 1180 μm, Sigma-Aldrich G1152) and 10 μL of Proteinase K at 20 mg/mL were added to the tubes. The samples were bead-beaten at 4 m/s for 20 s and incubated at 55°C for 2 h, followed by 65°C for 15 min. After this step, 62.5 μL of KOAc at 5 M were pipetted into the tubes and incubated on ice for 30 min. After spinning the tubes at 25,000 *g* for 15 min at room temperature, the supernatant was transferred into new 1.5 mL sterile microcentrifuge tubes and 0.8 vol. isopropanol was added to precipitate DNA. The solutions were left at room temperature for 15 min and centrifuged again at 25,000 *g* for 15 min. After removing the supernatant, the precipitate was washed with 100 μL of 70% ethanol, centrifuged at 25,000 *g* for 3 min, air-dried and resuspended overnight in 25 μL MilliQ water.

The same DNA suspensions were used to amplify the 16S rRNA gene and ITS2 marker. Variable regions V5 to V6 of the 16S rRNA gene were amplified using the forward primer 784F [5′-TCGTCGGCAGCGTCAGATGTGTATAAGAGACAGAGGATTAGATACCCTGGTA-3′] and reverse primer 1061R [5′-GTCTCGTGGGCTCGGAGATGTGTATAAGAGACAGCRRCACGAGCTGACGAC-3′] (Andersson et al., 2008; Röthig et al., 2016). The Symbiodiniaceae ITS2 region was amplified with the specific primer pair ITS2F [5′-TCGTCGGCAGCGTCAGATGTGTATAAGAGACAGGTGAATTGCAGAACTCCGTG-3′] and ITS2R [5′-GTCTCGTGGGCTCGGAGATGTGTATAAGAGACAGCCTCCGCTTACTTATATGCTT-3′] (Boulotte et al., 2016). The underlined segments represent Illumina adapter overhangs (Illumina, San Diego, CA, United States). The PCRs were conducted in 10 μL triplicates using the AmpliTaq Gold 360 Master Mix and 0.4 μM of each primer. The amplification cycles were: 95°C for 10 min; 30 cycles each at 95°C for 30 s, 57°C for 1 min, 72°C for 30 s; a final extension at 72°C for 7 min. The PCR triplicates for each template were pooled and sent to Ramaciotti Centre for Genomics (UNSW, Sydney) for library preparation and sequencing on the Illumina MiSeq system with 2 × 300 bp paired-end reads.

In addition to processing the samples, six blank DNA extractions and three no-template PCRs were performed and sequenced to check for laboratory contamination.

### Bioinformatics

Both the 16S rRNA gene partial sequences and ITS2 region sequences were processed using the QIIME 2 pipeline version 2017.10 (Caporaso et al., 2010; QIIME 2 Development Team, 2017a). Plugin demux (QIIME 2 Development Team, 2017b) was used to visualize interactive quality plots and check read quality. Plugin DADA2 (Callahan et al., 2016) was subsequently applied to remove primers, truncate poor-quality bases based on the interactive plots, dereplicate, identify chimeras and to merge paired-end reads. Commands included in the plugin feature-table (McDonald et al., 2012) enabled generation of summary statistics of sequences associated with the samples. For 16S rRNA gene sequences, a Naïve Bayes Classifier was trained with the feature-classifier plugin (QIIME 2 Development Team, 2017d) using the 16S rRNA gene database at 99% similarity of the SILVA 128 QIIME release (Quast et al., 2013) and based on the 784F/1061R primer pair. A phylogenetic tree for further downstream analyses was created with the plugins alignment (Katoh and Standley, 2013) and phylogeny (Price et al., 2010). Finally, the taxa plugin (QIIME 2 Development Team, 2017c) allowed to filter mitochondria and chloroplast sequences, as well as to visualize taxonomic bar plots and generate tables with absolute read counts of all taxa for each sample. For ITS2 sequences, taxonomic assignment was performed using the Arif et al. (2014) ITS2 database with the method vsearch (Rognes et al., 2016), at 97% sequence similarity level. The biom tables containing taxonomic counts, the metadata and phylogenetic trees were imported into R (R Core Team, 2018) for statistical analyses.

### Statistical Analyses

Exploratory and statistical analyses were performed at the amplicon sequence variant (ASV) level, a higher-resolution equivalent of the operational taxonomic unit (OTU) (Callahan et al., 2017). Taxa for which the minimum overall relative abundance was lower than 10^-5^ were filtered out of the dataset in order to mitigate the generation of spurious sequences (Bokulich et al., 2013). To account for the variability in the number of reads per sample, data counts were rarefied to an even depth, corresponding to the minimum number of reads across samples.

Recruit survival rates were modeled using generalized linear models with binomial distribution, and linear contrasts were used to test for differences between the treatments. Alpha diversity as a measure for richness was computed using Chao and Shannon α-diversity indices (Lande, 1996; Legendre and Legendre, 1998). To better understand differences in richness between coral species and treatments, generalized linear models with gamma distributions were fitted, and linear contrasts were used to test differences in richness. Differences in community composition (β-diversity, Anderson et al., 2006) were computed using Bray–Curtis dissimilarity matrices and tested via permutational multivariate analysis of variance (PERMANOVA, Anderson, 2001). Variation in community composition among samples was visualized using Principal Coordinate Analysis (PCoA) (Legendre and Legendre, 1998). A test for homogeneity of multivariate dispersions (Anderson, 2006) was used to check for homogeneity of variances and pairwise comparisons were performed between groups using a single-step (Scheffé, 1953) and the Benjamin and Hochberg corrections for multiple testing (Benjamini and Hochberg, 1995). In addition, we performed differential pairwise abundance comparisons using unrarefied data based on generalized linear models with negative binomial distributions as described in Love et al. (2014), to identify taxa for which there was a significant logarithmic fold change (LFC) in abundance between groups. In addition, random Forest classification was used to understand feature importance of the bacterial community (Breiman, 2001).

All statistical analyses were conducted using R version 3.3.2 (R Core Team, 2018) and packages phyloseq (McMurdie and Holmes, 2013), vegan (Oksanen et al., 2016), DESeq2 (Love et al., 2014), tidyverse (Wickham, 2017), randomForest (Liaw and Wiener, 2002), RVAideMemoire (Herve, 2018) and multcomp (Hothorn et al., 2008) and ggplot2 (Wickham, 2009).

## Results

### Survival of Coral Recruits

**FIGURE 2:**
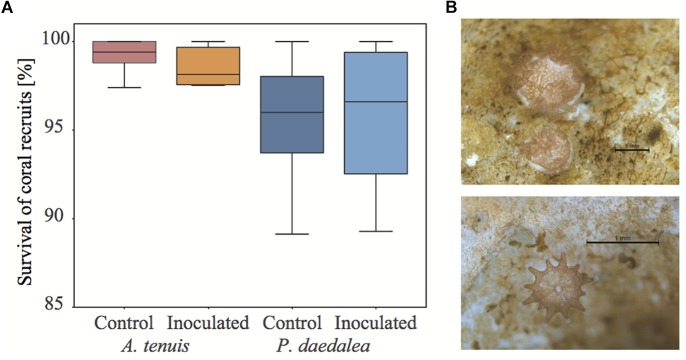
**(A)** Survival of control and inoculated *A. tenuis* and *P. daedalea* recruits at the end of the experiment (42 days post-spawning, after seven inoculations) as compared to the start of the experiment (16 days post-spawning, before any inoculation). **(B)** Representative pictures of *A. tenuis* (top) and *P. daedalea* recruits (bottom) at four weeks post-settlement. Black scale bar is 1 mm.

Following settlement, 962 *A.* *tenuis* and 566 *P. daedalea* recruits were distributed across the 12 experimental tanks. Thus, 80 *A. tenuis* and 47 *P. daedalea* recruits were randomized in each tank at the start of the experiment. The average survival of the 4-week old recruits was high (*Acro*_Ctrl_: 99.2%, *Acro*_Inoc_: 98.6%, *Platy*_Ctrl_: 95.5%, *Platy*_Inoc_: 95.7%, Figure 2A) with no significant difference in survival across species or treatment (for all pairwise contrasts: *p* > 0.9). However, survival appeared more variable among tanks for *P. daedalea* recruits than for *A. tenuis* recruits. Recruits of both coral species were well pigmented, meaning that they successfully established symbiosis with *C. goreaui* (Figure 2B).

### Sequence Data Statistics and Diversity

After removal of rare sequences (minimum total relative abundance < 10^-5^), a total of 1,162,570 high quality 16S rRNA gene reads were obtained for 53 samples, representing 2,026 ASVs belonging to 196 bacterial families. The number of reads per sample varied between 10,290 and 34,180 (Supplementary Figure S1). Coral recruits and water samples were respectively associated with a total of 1,362 ASVs and 960 ASVs, sharing a total of 417 ASVs.

Samples were rarefied at 10,290 reads to account for the variability in sequencing effort and rarefaction curves confirmed that this depth was sufficient to reflect the diversity present in the samples. Rarefaction curves displayed an asymptote before reaching the set threshold of 10,290 reads (Supplementary Figure S2). Moreover, in all sample groups after rarefaction, the similarity between the observed and expected richness (Chao index) was greater than 96%. For each sample category, the observed and Chao diversity index, evenness and Shannon α-diversity index estimates are reported in Table 2 together with their standard deviations (s.d).

**Table 2:** Overview of the number of samples and corresponding diversity indices for 16S rRNA sequences (average ± s.d.).

Sampling group		No of samples	Observed Richness	Chao diversity index estimate	Evenness	Shannon α-diversity index
*A. tenuis*	Control	6	307 ± 44	314 ± 47	0.81 ± 0.03	4.61 ± 0.21
	Inoculated	6	254 ± 51	256 ± 52	0.77 ± 0.04	4.27 ± 0.38
*P. daedalea*	Control	6	391 ± 32	401 ± 35	0.81 ± 0.02	4.83 ± 0.14
	Inoculated	6	333 ± 34	336 ± 37	0.79 ± 0.02	4.62 ± 0.16
Water	Control	6	316 ± 21	330 ± 20	0.72 ± 0.02	4.13 ± 0.12
	Inoculated	6	285 ± 20	293 ± 21	0.74 ± 0.02	4.17 ± 0.10
*C. goreaui* culture		1	71	69	0.67	2.86
Bacterial cocktail (7 inoculations)		7	8 ± 1	8 ± 1	0.82 ± 0.04	1.75 ± 0.05
Blank DNA extractions		6	47 ± 36	47 ± 36	0.58 ± 0.07	2.16 ± 0.36
No template PCRs		3	18 ± 7	18 ± 7	0.57 ± 0.02	1.65 ± 0.29


**FIGURE 3:**
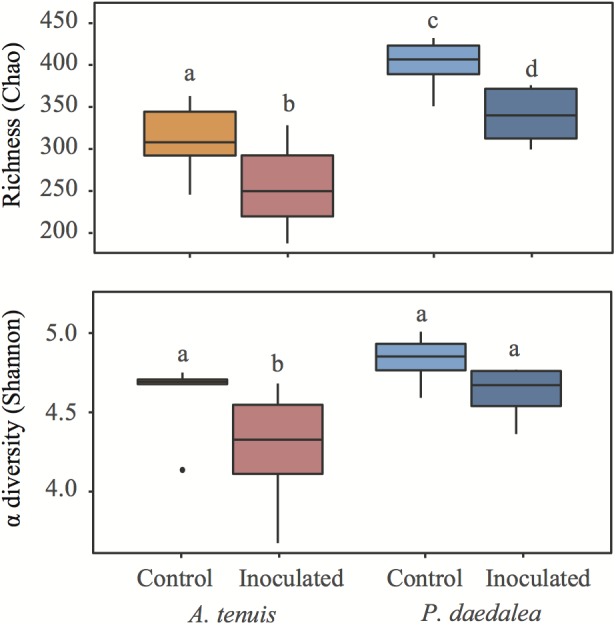
Boxplots representing richness based on Chao and Shannon α-diversity indices in coral recruits at the end of the experiment. Different letters denote groups with significantly different richness or Shannon α-diversity index (*p* < 0.05). Within each graph, groups with the same letter do not have a statistically significant difference in α-diversity.

*Platygyra daedalea* recruits displayed significantly higher bacterial species richness than *A. tenuis* recruits for both control and inoculated treatments (Figure 3; *z*_Ctrl_ = 2.905, *p* < 0.01; *z*_Inoc_ = 3.247, *p* < 0.001). Within each coral species, the inoculated recruits had significantly lower bacterial species richness than the control recruits (*z*_Acro_ = -2.439, *p* = 0.015; *z*_Platy_ = -2.091, *p* = 0.036). The Shannon α-diversity index was higher for *P. daedalea*, although the difference was only significant in the inoculated treatment (Figure 3; *z*_Ctrl_ = -1.497, *p* = 0.134; *z*_Inoc_ = -2.521, *p* = 0.012); Shannon α-diversity index was significantly lower in inoculated *A. tenuis* compared to inoculated *P. daedalea* (*z*_Acro_ = 2.422, *p* = 0.015; *z*_Platy_ = 1.398, *p* = 0.162).

The Symbiodiniaceae composition of coral recruits was analyzed and compared with the pure culture that was inoculated to the recruits at the start of the experiment (Supplementary Figure S3). Both *A. tenuis* and *P. daedalea* juveniles exclusively harbored *C. goreaui* (formerly known as clade C; LaJeunesse et al., 2018) sequence types that were also recovered from the monoclonal cultures used for inoculation. Since more than one *C.* *goreaui* sequence variant was found, we conclude that intragenomic variation exists within the inoculated *C. goreaui* culture. Intragenomic variation is common and widespread within the Symbiodiniaceae (Wilkinson et al., 2015, 2018). The *C. goreaui* culture also comprised 74 bacterial ASVs, largely dominated by Rhodobacteraceae and Flammeovirgaceae (Supplementary Figure S4).

### Sequencing Control Samples

DNA contamination originating from laboratory reagents and sample handling, as well as biases occurring during PCR amplification and sequencing have often been reported to distort the results obtained via metabarcoding (Salter et al., 2014; Fouhy et al., 2016). The bacterial community composition data from coral recruits, water collected from the aquaria, and negative controls (i.e., blank extractions and no-template PCR products), were subjected to PERMANOVA analysis, which confirmed that corals and water samples hosted significantly different bacterial communities from negative controls. Bacterial communities in water and coral also differed significantly from one another (Supplementary Table S1 and Supplementary Figure S5).

A total of 237 out of the 2,026 detected ASVs were present in the negative control samples, with four ASVs belonging to the genera *Burkholderia-Paraburkholderia, Ralstonia* and an unidentified Oxalobacteraceae dominating the blank (73.2%) and no-template samples (73.4%), (Supplementary Table S2). As these four ASVs had a 10–100 fold lower abundance in the test samples, it is reasonable to assume that their presence was due to laboratory or reagent contamination. As recommended (Lee et al., 2015), these four ASVs were removed from the dataset prior to further analyses.

The use of mock communities has been highly recommended to assist in estimating biases in the overall metabarcoding process (e.g., PCR, sequencing and bioinformatics) (Yeh et al., 2018). In this study, the seven inocula comprising seven different bacterial species were used as mock communities. The cultures were quantified by OD_660_ and from this, their relative abundances (in terms of cells/mL) were estimated. These estimates were similar, although not identical, to the organismal relative abundances obtained by metabarcoding data analysis (Supplementary Figure S6). The ASVs with 100% sequence identity to the seven bacterial cultures represented more than 99% of the counts in each inoculum. We thus conclude there were minimal biases in the metabarcoding process. These seven ASVs will henceforth be referred to as “ASV1” to “ASV7” (being *Acinetobacter*, *Bacterioplanes*, *Marinobacter*, *Paracoccus*, *Pseudoalteromonas*, *Pseudovibrio*, *Vibrio*, respectively).

### Influence of Bacterial Inoculation and Host Species on Coral-Associated Bacterial Communities

**FIGURE 4:**
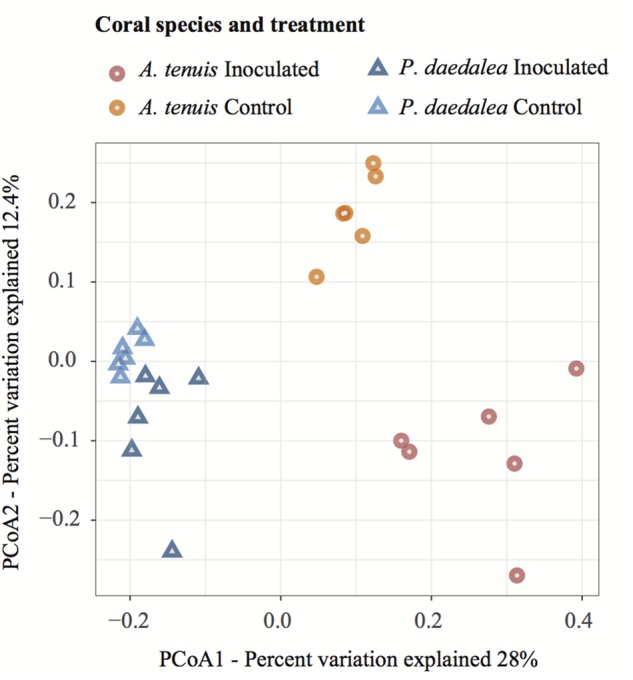
PCoA visualization using the Bray–Curtis dissimilarity measurement separating samples by treatment and host species. Samples were collected at the end of the experiment (day 42) following the seven bacterial inoculations.

In all treatments, *A. tenuis* and *P. daedalea* recruits were dominated by the bacterial class Alphaproteobacteria, followed by Gammaproteobacteria (Supplementary Figure S7). PCoA based on ASV data showed that coral samples clustered according to host species and treatment (inoculation vs. control; Figure 4). On inspection of the multivariate homogeneity of group variances, *A. tenuis* recruits were characterized by a higher dispersion than *P. daedalea* recruits (PERMDISP with 999 permutations: *F* = 14.72, *p* = 0.001).

The bacterial microbiomes harbored by *A. tenuis* and *P. daedalea* recruits were significantly different from one another (for both treatments), and the difference in community composition between inoculated and control recruits of the same species was also statistically significant (PERMANOVA with 999 permutations: *F*_Treatment_ = 3.6279, *p* = 0.001; *F*_Species_ = 8.48, *p* = 0.001). After checking for homogeneity of variances among all four groups (PERMDISP with 999 permutations: *F* = 2.77, *p* = 0.066), pairwise comparisons with correction for multiple testing confirmed that the treatment had a significant effect within each coral species (*A. tenuis* Inoc vs. Ctrl: *p* = 0.002, *P. daedalea* Inoc vs. Ctrl: *p* = 0.002).

**FIGURE 5:**
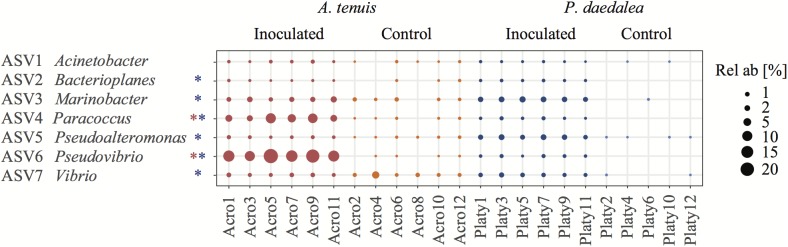
Bubble plot representing the relative abundance within each sample of the seven bacterial ASVs used for inoculation (i.e., having a 100% sequence identity to the present in the inocula). The asterisks next to the bacterial names indicate that this ASV had a significantly different LFC in abundance between control and inoculated recruits. Coral samples were collected at the end of the experiment (day 42) following the seven bacterial inoculations. Red refers to *A. tenuis* while blue refers to *P. daedalea*. Rel ab, relative abundance.

**FIGURE 6:**
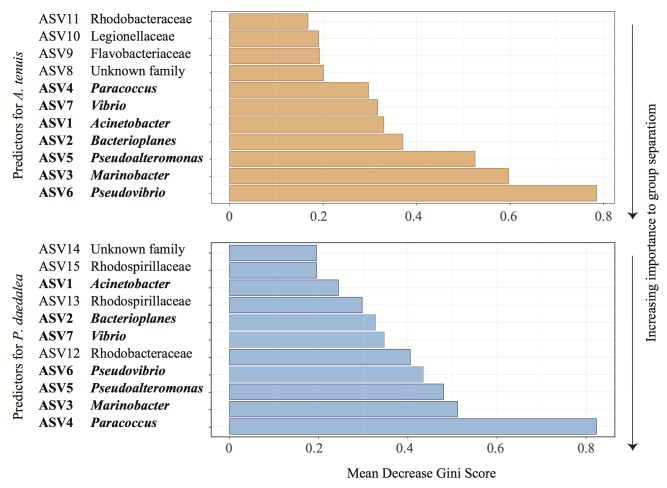
Representative output of the random Forest classification with 100 iterations to identify the most important predictor ASVs in *A. tenuis* (orange) and *P. daedalea* (blue). The genus or family (when known) of each ASV is specified. Names written in bold represent the inoculated ASVs.

The relative abundances of two and six bacterial strains used in the inocula were significantly greater in the inoculated compared to the control recruits for *A. tenuis* and *P. daedalea*, respectively (Figure 5). For each coral species, a differential abundance analysis was performed on the non-rarefied dataset to identify ASVs for which there is a significant LFC in abundance between inoculated and control recruits. Using a significance level of α = 0.05, only bacteria contained in the inocula were reported to have a significantly different LFC between treatments (two for *A. tenuis* and six for *P. daedalea*, Table 3). Random Forest analyses performed on the microbiome data of each coral species corroborated the results obtained with differential abundance analysis. In *A. tenuis* and *P. daedalea*, ASVs matching the seven inoculated ones were classified as the most important predictors for the discrimination of inoculated and control recruits (Figure 6). In this analysis, several bacterial taxa (such as members of Rhodobacteraceae and Rhodospirillaceae) not present in the inocula were also classified as important predictors for separating control and inoculated recruits in each coral species (Figure 6).

**Table 3:** Bacteria having a significantly different LFC in abundance between inoculated and control recruits.

Coral host	ASV	Bacterial genus	Log_2_ Fold Change	*p_Adj_*
*A. tenuis*	ASV4	*Paracoccus*	5.69	2.4⋅10^-4^
	ASV6	*Pseudovibrio*	8.49	1.57⋅10^-9^
*P. daedalea*	ASV2	*Bacterioplanes*	7.70	8.85⋅10^-6^
	ASV3	*Marinobacter*	5.84	6.59⋅10^-3^
	ASV4	*Paracoccus*	8.03	5.25⋅10^-6^
	ASV5	*Pseudoalteromonas*	5.48	2.75⋅10^-4^
	ASV6	*Pseudovibrio*	8.19	4.29⋅10^-6^
	ASV7	*Vibrio*	5.11	1.40⋅10^-2^


*Results of the differential abundance analysis are reported for the Wald test and parametric fit.*

**FIGURE 7:**
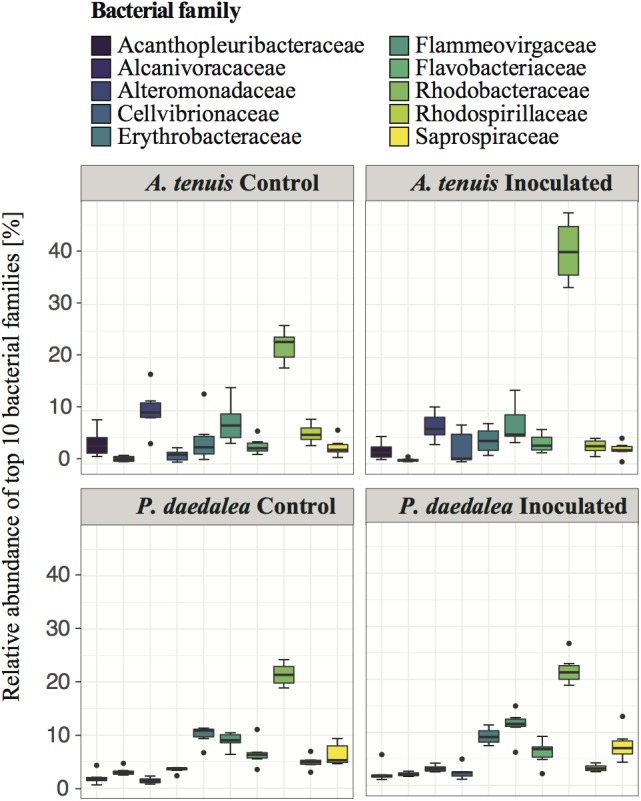
Relative abundance of the 10 most prevalent bacterial families across all coral samples collected at the end of the experiment (day 42).

Despite being reared in a common environment, *A. tenuis* and *P. daedalea* recruits overall developed distinct bacterial communities. The most abundant bacterial families across coral samples showed some degree of variability between *A. tenuis* and *P. daedalea* samples, as well as between inoculated and control recruits (Figure 7). For instance, family Rhodobacteraceae (to which the inocula strains *Paracoccus* and *Pseudovibrio* belong) was highly abundant in *A. tenuis* and particularly so in the inoculated recruits. Family Alteromonadaceae was also more abundant in *A. tenuis* compared to *P. daedalea* (Figure 7).

**FIGURE 8:**
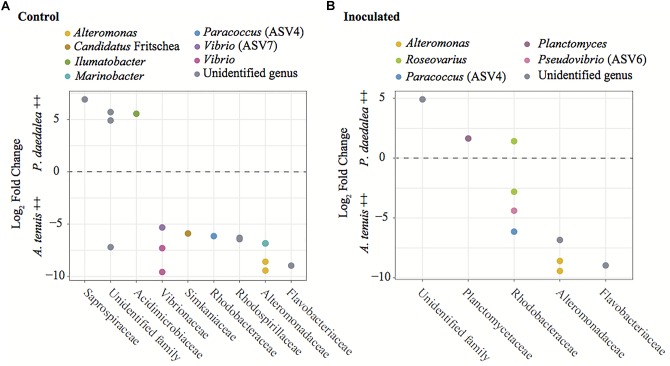
Results from the differential abundance analysis with significance level α = 0.01, expressed as Log_2_ Fold Change for the comparison between *A. tenuis* and *P. daedalea* recruits within each treatment: **(A)** control recruits and **(B)** inoculated recruits. Each dot represents one ASV (including ASVs 4, 6, and 7 from the inocula), here identified at the genus level when possible. Positive values represent ASVs proportionally more abundant in *P. daedalea*, while negative values are ASVs proportionally more abundant in *A. tenuis*.

A differential abundance analysis was performed on the coral recruits within each treatment to identify the ASVs for which the relative abundance significantly differed between *A. tenuis* and *P. daedalea* (Figure 8). ASVs belonging to Alteromonadaceae (two different *Alteromonas* ASVs) were found significantly more abundant in *A. tenuis* for both control and inoculated corals. Rhodobacteraceae [including *Paracoccus* (ASV4), *Pseudovibrio* (ASV6) and a *Roseovarius* (ASV)] were significantly more abundant in inoculated *A. tenuis* compared to inoculated *P. daedalea*. In the control treatments, Vibrionaceae (three *Vibrio* ASVs including ASV7) were more abundant in *A. tenuis* than in *P. daedalea.*

Some assignable families exclusively appeared in one coral species (5 in *A. tenuis* and 10 in *P. daedalea*) or in the water (39 families) (Supplementary Figure S8). For example, members of the families Hahellaceae (all belonging to the genus *Endozoicomonas*), Comamonadaceae, Colwelliaceae and Fibrobacteraceae were detected in *A. tenuis* but not in *P. daedalea*, while Nitrosomonadaceae, Polyangiaceae, Parachlamydiaceae, Oxalobacteraceae were detected in *P. daedalea* and absent from *A. tenuis*.

## Discussion

### Coral Host Factors Influence the Microbiome

While some studies report that coral-associated bacterial communities depend on environmental factors and geographical location (Littman et al., 2009b; Lee et al., 2012; Pantos et al., 2015; Hester et al., 2016), other reports indicate that corals harbor species-specific bacterial assemblages (Rohwer et al., 2002; Rohwer and Kelley, 2004; Bourne et al., 2008; Littman et al., 2009b; Tremblay et al., 2010; Apprill et al., 2012; Morrow et al., 2012; Ainsworth et al., 2015; Zhang et al., 2015; Chu and Vollmer, 2016). The observed differences in bacterial communities retrieved from corals versus those from the surrounding water column are compelling evidence that coral-bacteria associations are non-random and subject to selective mechanisms (Sunagawa et al., 2010; Sweet et al., 2010). Emerging data indicate both environmental and host factors are drivers of coral-associated microbial community composition (Kvennefors et al., 2010; Kelly et al., 2014; Zhang et al., 2015; Frade et al., 2016), and our study provides strong support for a role of host factors in shaping the composition of the microbiome. At the sampling time point, bacterial communities of the two coral host species cultured in the same aquaria since the early recruit stage differed in terms of α-diversity (bacterial communities in *P. daedalea* were richer than those in *A. tenuis*, Figure 2), β-diversity (there was more variability across samples in *A. tenuis* than in *P. daedalea*, Figure 3), and in the relative abundance (Figure 7) and presence/absence of particular bacterial taxa (Supplementary Figure S8). In addition to harboring distinct bacterial communities, the two corals responded differently to the inoculations in that the degree of shift away from the bacterial community harbored by the control recruits was larger in *A. tenuis* than in *P. daedalea* (Figure 3). The intrinsically higher microbial diversity in *P. daedalea* might have buffered the perturbation introduced by the inocula, as empirical studies and mathematical models support the notion that diverse ecosystems are more resistant to invasion than systems with lower diversity (Levine and D’Antonio, 1999). The bacterial profiles of control *A. tenuis* and *P. daedalea* recruits are challenging to compare with already published data. Previous studies on *A. tenuis* also report dominance of Alpha- and Gammaproteobacteria (Supplementary Figure S7), but with variable proportions (Littman et al., 2009b; Ceh et al., 2011). Only limited information exists on prokaryotic communities associated with *P. daedalea*; one study focused on culturable Actinobacteria (Mahmoud and Kalendar, 2016), while another work reported a *P. daedalea* sample with a high abundance of Alpha- and Gammaproteobacteria, mostly represented by *Endozoicomonas* (Liang et al., 2017). Different species of adult corals have been reported to maintain distinct microbiomes when reared in identical environments (Sweet et al., 2013), and our study shows that host factors already play a role from early ontogeny. Importantly, our data were not confounded by the Symbiodiniaceae species, which can influence bacterial community composition in young corals (Littman et al., 2009a), as both species were inoculated with the same monoclonal *C. goreaui* culture.

### *A. tenuis* and *P. daedalea* Recruits Responded Differently to the Inocula

Some of the ASVs corresponding to the inoculated bacterial strains were more significantly abundant in the inoculated compared to the control coral recruits (Figure 5). The two corals, *A. tenuis* and *P. daedalea*, may exhibit specific preferences for certain bacterial taxa and possess different selection mechanisms to shape their bacterial communities. Potential probiotics for corals might therefore need to be tailored to particular bacterial species in order to achieve optimal uptake. Interestingly, the same two ASVs significantly enriched in inoculated *A. tenuis* (ASV4 – *Paracoccus* and ASV6 – *Pseudovibrio*) were also characterized by the highest LFC in *P. daedalea* (Table 3). ASV4 and ASV6 both belong to class Alphaproteobacteria, which was the dominant bacterial class in all coral recruits in this study, while the five other ASVs used in the inocula are Gammaproteobacteria.

*Acropora tenuis* is a fast growing and branching coral, while *P. daedalea* is slow growing, massive coral with a thick tissue layer, which provides more shading to the *in hospite* algal symbionts and energy reserves for the host (Loya et al., 2001; Putnam et al., 2017). Such contrasting morphological and physiological properties might create microhabitats suitable for different types of microbes and also dictate requirements for specific microbial functions. It is therefore not surprising that *A. tenuis* and *P. daedalea* established associations with different microbial communities over the course of this experiment. Field surveys have reported that branching and massive corals naturally tend to associate with distinct bacterial communities, with higher α-diversities generally observed in massive species (Liang et al., 2017).

It is possible the young *A. tenuis* and *P. daedalea* had already acquired a subset of bacteria from the water surrounding parental colonies following gamete release and/or from the water in which larvae and recruits were reared. Despite being filtered with 0.4 μm pore-sized membranes, the flow-through water in the experimental system contained some bacteria (as shown by the 16S rRNA gene sequences found in water samples). However, the water-associated microbial communities were considerably divergent from the coral microbiota (Supplementary Figure S5), which emphasizes the ability of corals to select certain bacteria from the environment. Alternatively, adult *A. tenuis* and *P. daedalea* may have vertically transmitted some bacterial symbionts to their offspring prior to spawning. Evidence to date indicates that the bacterial transmission mode in broadcast spawning corals is variable (Sharp et al., 2010; Lema et al., 2014; Neave et al., 2016; Leite et al., 2017; Zhou et al., 2017).

### Effect of Bacterial Inoculation on Coral Recruit Microbiome

Bacterial community composition was not assessed at the start of the study due to a limited number of recruits available. It is possible that some bacteria were inherited from parental coral colonies and/or horizontally acquired from the environment. The latter includes bacteria present in the FSW entering the rearing tanks, as well as the bacteria found in the *C. goreaui* culture, which was administered to all recruits (Supplementary Figures S2, S4). However, knowledge of this baseline microbiome was not necessary to evaluate the effect of bacterial inoculation, since inoculated recruits were compared to control counterparts, which were treated in the same manner. By exposing coral recruits to a chosen bacterial consortium, we were able to significantly modify their microbiome (Figure 3). Several bacterial strains in the inocula (i.e., two in the case of *A. tenuis* and six for *P. daedalea*) were statistically significantly enriched in the inoculated recruits (Figure 5). Bacteria used in the inocula were also among the major ASVs driving the separation of bacterial communities associated with control and inoculated recruits (Table 3). Effective inoculations were thus not precluded by using bacteria isolated from non-coral organisms, which demonstrates a degree of flexibility in the coral microbiome. Altogether, this study supports the proof-of-concept for the feasibility of manipulating coral-associated prokaryotes (dos Santos et al., 2015; Rosado et al., 2018).

### Knowledge Gaps, Experimental Shortcomings and Directions for Future Research

To gain more insights into the efficacy of probiotics for coral reef restoration and conservation, future research should target several aspects not covered in the present study. First, an optimal inoculation regime should be developed through the determination of suitable inoculation frequency and bacterial cell density. In the present work, the seven inoculations at 10^5^–10^6^ cells/mL arbitrarily followed a 3-4-3-4 days pattern, as no standard procedure has yet been established. Alternative strategies have also yielded effective outcomes, such taking coral fragments out of the water and inoculating them with 1 mL (10^7^ cells/mL) twice 5 days apart (Rosado et al., 2018). Nonetheless, systematic experiments are required to determine optimal inoculation protocols, by using the same bacterial taxa, coral species and culture conditions while varying bacterial cell density and/or frequency of inoculum administration.

This experiment was conducted over a short timescale (4 weeks), which did not allow the long-term stability of coral-bacterial associations to be evaluated. It is not clear whether the dosed bacteria would be retained over time, especially when no further bacteria are delivered. Also, shifts in the coral microbiome following transfer between field and aquaria settings have been reported (Pratte et al., 2015; Röthig et al., 2017), which could challenge the implementation of microbiome manipulation strategies if the goal is to achieve long-term changes in the coral-associated microbiome. The coral microbiome is dynamic (Mouchka et al., 2010; Sweet and Bulling, 2017), especially in early life stages (Littman et al., 2009a; Zhou et al., 2017; Epstein et al., 2019a). As recruits develop and grow, the complex microbial communities associated with juveniles shift toward less diverse adult microbiomes. Due to these natural processes, the impact of bacterial inoculations during early life stages might diminish over time, but this needs to be tested. In addition to life stage, numerous other biotic and abiotic factors may influence the coral microbiome, such as abundance and changes in Symbiodiniaceae species, coral disease, and water parameters of salinity, temperature, pH and nutrient levels (reviewed in Hernandez-Agreda et al., 2016b). The capacity for sustained bacteria-coral associations following inoculation needs to be studied beyond laboratory-controlled conditions to understand the impact of these fluctuating factors.

An additional important piece of knowledge lacking from our study is the growth and viability of the bacterial cells used to prepare the consortium prior to coral inoculation. The possibility that some of the 16S rRNA gene sequences recovered from the recruits originated from dead organisms or residual DNA adhering to the polyps can thus not be excluded. However, our results support a true change in the coral microbiomes rather than residual DNA on the samples being responsible for the observed changes. Firstly, *A. tenuis* and *P. daedalea* exhibited distinct enrichments of ASVs corresponding to the bacterial strains used in the inocula. Secondly, not all strains were significantly enriched in inoculated recruits (two in the case of *A. tenuis* and six in the case of *P. daedalea* – Figure 5). Differential enrichment according to host species and failure to detect a statistically significant higher abundance for all seven strains in inoculated recruits indicate a preferential uptake of bacterial groups by the corals. Lastly, based on random Forest classification, ASVs other than the ones present in the inocula were identified as important predictors to separate control and inoculated recruits (Figure 6). These patterns suggest a change in the host bacterial community composition that is promoted by the inoculation.

Assessing the location of the inoculated bacteria within the host will also be informative. In this study, the enrichment of the inoculated bacteria in the coral recruits could originate from internalized communities or from organisms adhering to the surface of the recruits. Bacteria within the mucus layer or the gastric cavity of polyps might be transient due the high variability that usually characterizes these microhabitats (Sweet et al., 2010; Thompson et al., 2015; Glasl et al., 2016). Moreover, it needs to be verified that inoculated bacteria are not simply taken up as food source, but rather become part of the coral microbiome. Stable partnerships have been suggested to occur between the coral host and several bacterial taxa occupying intracellular spaces (Ainsworth et al., 2015). These bacteria have been hypothesized to comprise the coral core microbiome, which is conserved across time and geographical location (Hernandez-Agreda et al., 2016a). Visualizing sections of inoculated corals via fluorescence *in situ* hybridization with probes targeting particular bacteria would allow localizing them when administered to specific host niches (Ainsworth et al., 2006; Wada et al., 2016). Even though a stable association seems more likely when microbes are endosymbiotic, consistently detecting the inoculated bacteria over time in any coral compartment would support the notion of a lasting partnership. Stably labeling cells with fluorescent proteins through genetic engineering as suggested by Pollock et al. (2015) would allow to precisely track the probiotic bacteria, as well as the offspring generated from their division.

### Practical Considerations

The application of probiotics to wild corals (or to captive corals aimed to be deployed to the field for reef restoration) requires further research into the possibility of scaling up such efforts. While industrial infrastructures to produce cultured bacterial consortia already exist, the latter need to be suitably delivered to corals. Inocula could be prepared by encapsulating bacteria into microscopic feed particles (Peixoto et al., 2017). Alternatively, probiotic bacteria could be administered through a heterotrophic food source such as the brine shrimp *Artemia*, which has already proven successful in spiny lobster larvae (Goulden et al., 2012). As the delivery of probiotics to entire reef systems could be unmanageable due to their large size as compared to available resources, efforts could be prioritized to most vulnerable or ecologically relevant sites.

Importantly, potential environmental impacts should also be strictly evaluated. It is indeed argued that coral reef ecosystems might be inadvertently harmed through manipulations that we cannot entirely predict or control (Sweet et al., 2017). For instance, disease agents such as pathogens or parasites could be transferred from captive systems to the natural environment and impact the native fauna (Sweet et al., 2017). Moreover, potential probiotic bacteria (such as *Vibrio*) could be converted into pathogens if they acquired the right virulence genes (Bruto et al., 2017). This would be particularly problematic if probiotics are administered at large cell numbers. In general, an overabundance of certain bacteria in the wild could have unintended effects on the ecosystem. Therefore, future research should focus on understanding the risk of probiotics before releasing microbes into open reefs (National Academies of Sciences Engineering, and Medicine, 2019). It has already been advocated that rigorous scientific trials and risk/benefit analyses should be carried out prior to introducing any foreign microbial communities into the ocean (van Oppen et al., 2017).

## Conclusion

This study involved the co-culturing of two taxonomically divergent coral species and inoculating them with a bacterial cocktail generated in the laboratory. Despite sharing the same environment since a very early life stage, *A. tenuis* and *P. daedalea* recruits had distinct bacterial communities at the time of sampling, which exhibited different responses to the inocula. In the absence of confounding factors such as environmental parameters and algal symbiont type, our findings highlight that host factors play a noticeable role in shaping coral bacterial community composition. As the microbiomes of *A. tenuis* and *P. daedalea* changed in response to the inoculum, the bacteria present in the cocktail were identified as the ones driving the main differences between inoculated and control corals. The bacterial pool surrounding these young recruits therefore also influenced their microbiomes. The long-term maintenance and stability of inoculated bacteria in corals still need to be characterized. By demonstrating the feasibility to manipulate the coral microbiome in a laboratory setting, our results provide hope for the application of probiotics in coral reef conservation and restoration.

## Author Contributions

KD, MO, and LB developed the study design. KD conducted the experiment and collected the data. PM and KD performed the statistical analyses. KD wrote the manuscript with contributions from all authors. All authors read and approved the final manuscript.

## Conflict of Interest Statement

The authors declare that the research was conducted in the absence of any commercial or financial relationships that could be construed as a potential conflict of interest.
