# Supplementary material for: Experimental Inoculation of Coral Recruits With Marine Bacteria Indicates Scope for Microbiome Manipulation in Acropora tenuis and Platygyra daedalea
Source: Front Microbiol. 2019 Jul 24;10:1702. doi: 10.3389/fmicb.2019.01702 (PMC6668565; doi:10.3389/fmicb.2019.01702)
Supplement: Supplementary file 1 [file Table_1.docx]

Supplementary Material

**Experimental inoculation of coral recruits with marine bacteria indicates scope for microbiome manipulation in *Acropora tenuis* and *Platygyra daedalea***

**Katarina Damjanovic^*^, Madeleine J. H. van Oppen, Patricia Menéndez, Linda L. Blackall**

***Correspondence**: Katarina Damjanovic: kdamjanovic@student.unimelb.edu.au

**Supplementary figures and tables**


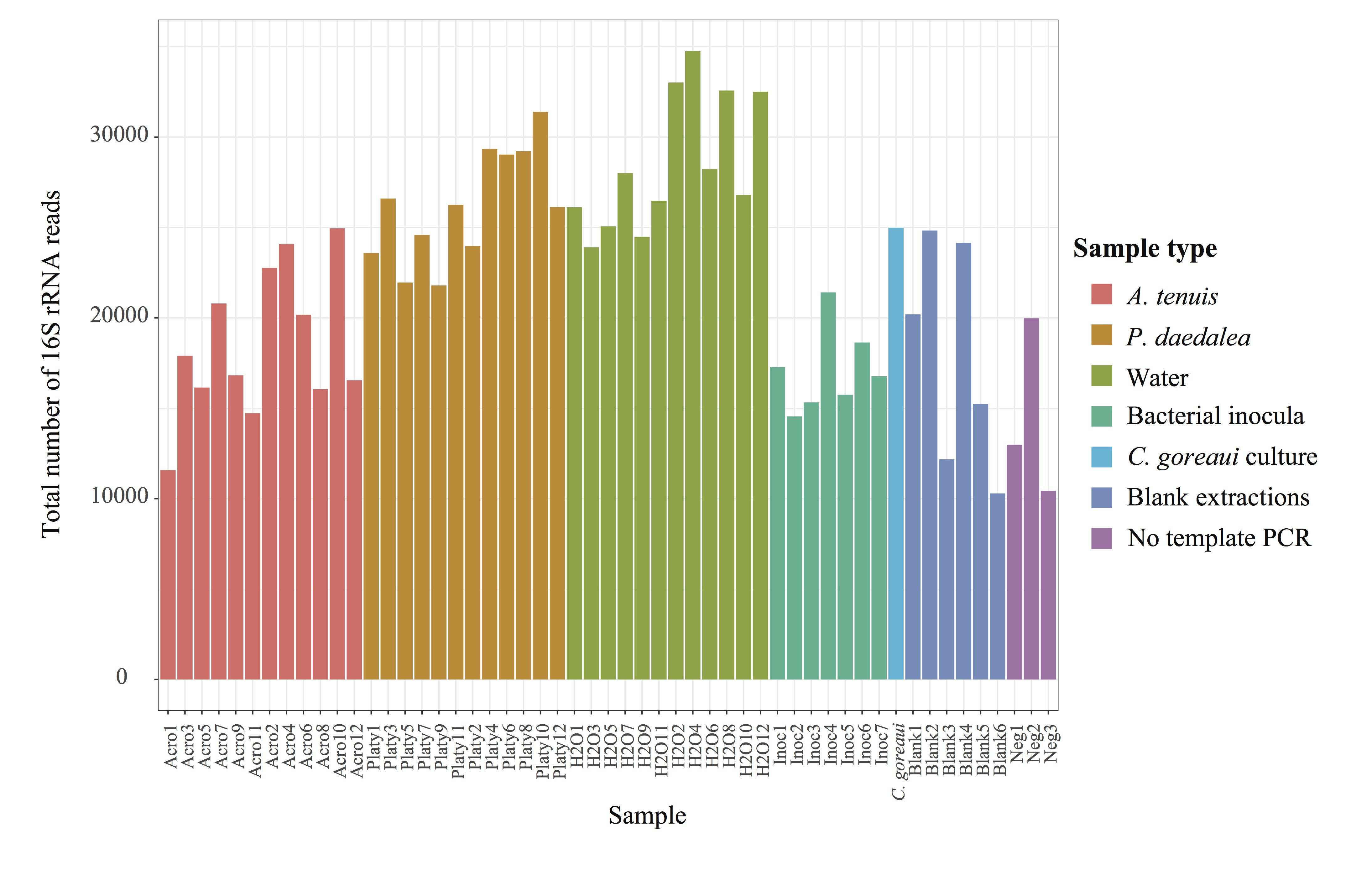


**Supplementary Figure 1.** Total number of 16S rRNA reads per sample after removal of rare taxa.

**
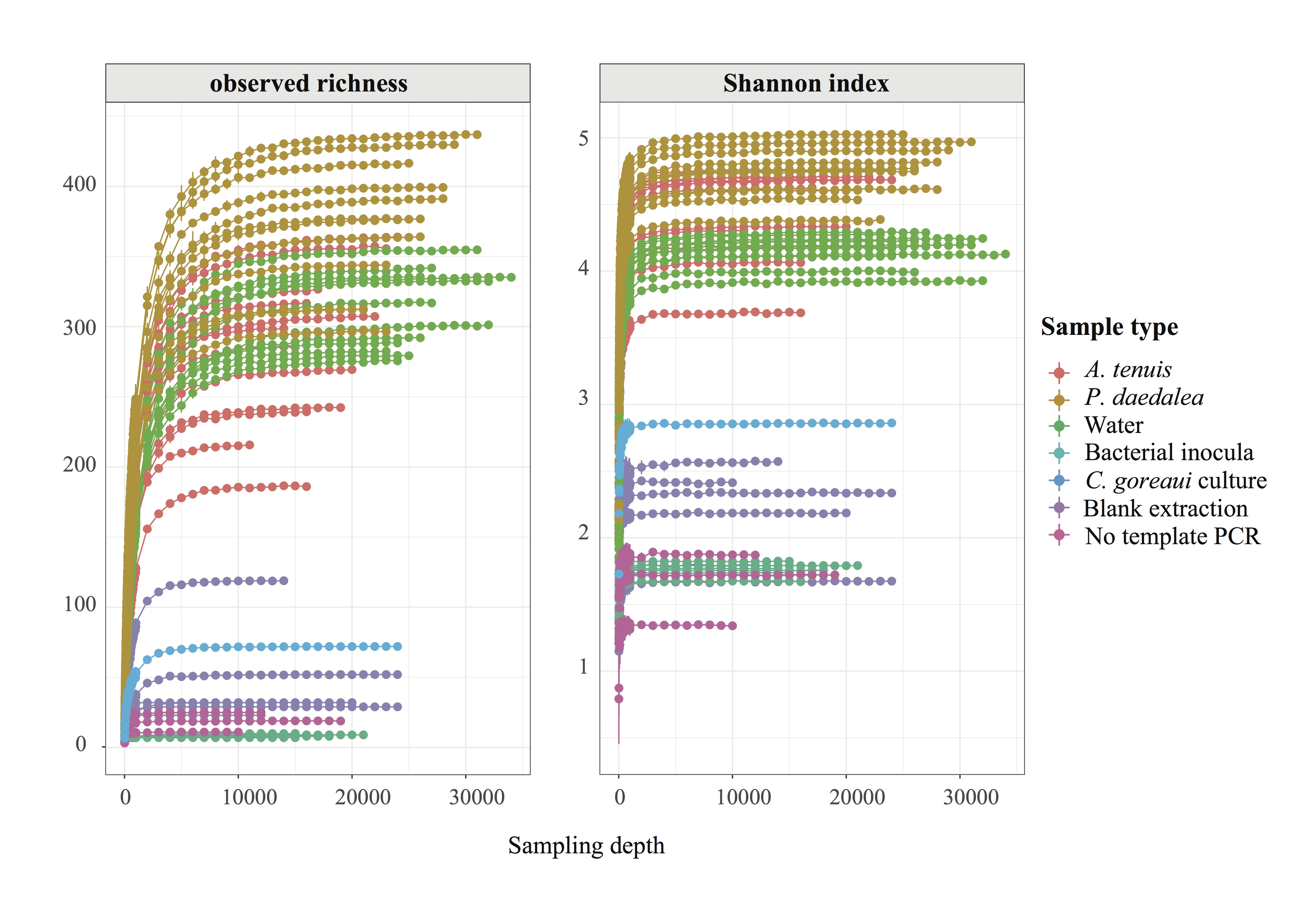
**

**Supplementary Figure 2.** ASV rarefaction curves of 16S rRNA sequences representing the diversity in all samples through the observed richness and Shannon index.

**
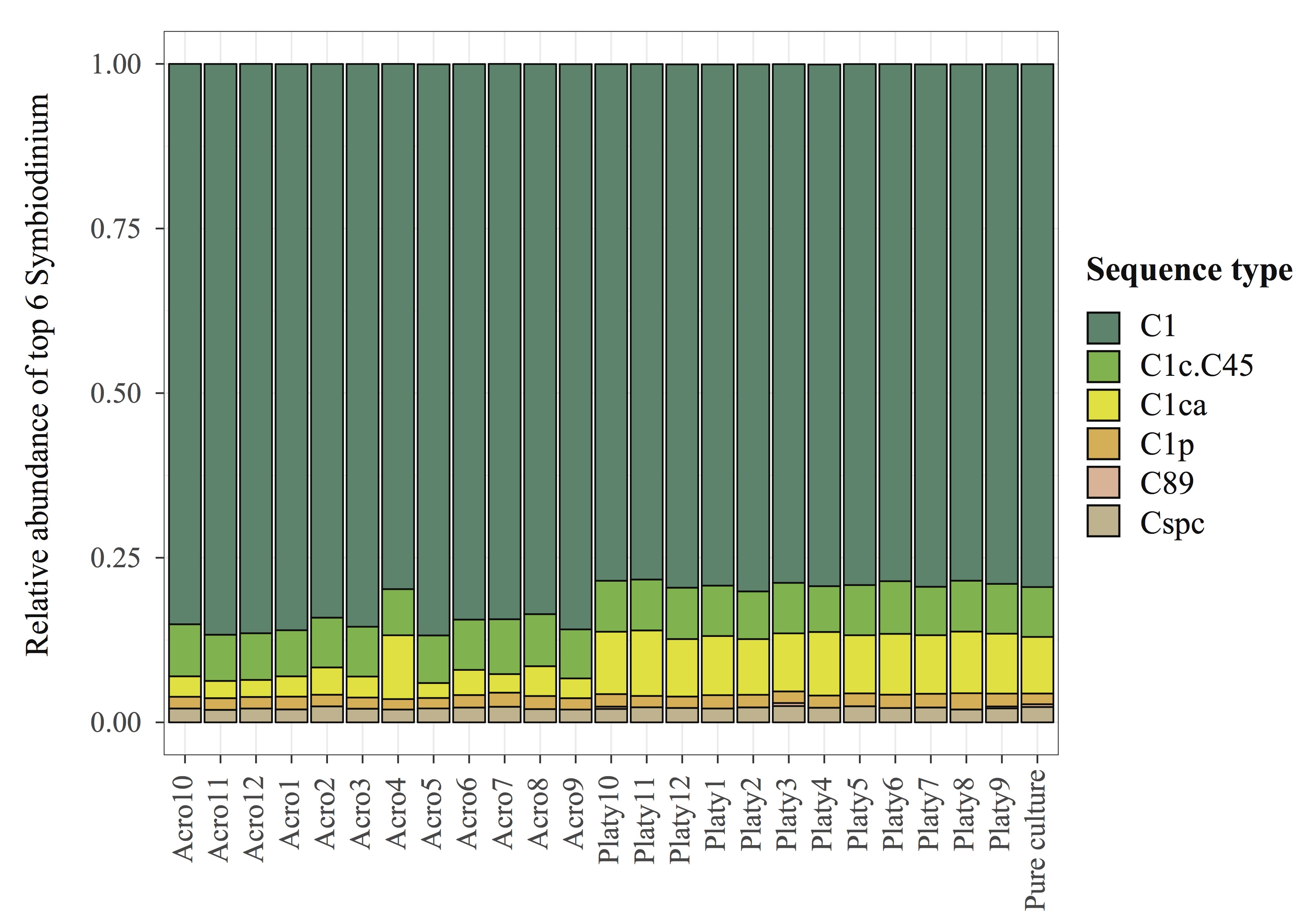
**

**Supplementary Figure 3.** Relative abundance of *Cladocopium* sp. sequence types associated with coral recruits and present in the monoclonal culture (*C. goreaui*; formerly C1) used for inoculation.

**Supplementary Figure 4.** Relative abundance of the 25 most prevalent bacterial families detected in the *C. goreaui* monoclonal culture, in which 74 ASVs were present in total. The ASVs comprised in these 25 families represented 99.07% of the total bacterial abundance in this sample.

| **Supplementary Table 1** PERMANOVA results for the comparison between coral, water and negative controls (blank DNA extractions and no-template PCR products) | | |
| --- | --- | --- |
| Groups compared | F | *p* |
|  |  |  |
| Corals - Water | 50.5 | < 0.001 |
|  |  |  |
| Corals - Negatives | 22.555 | < 0.001 |
|  |  |  |
| Water - Negatives | 26.795 | < 0.001 |


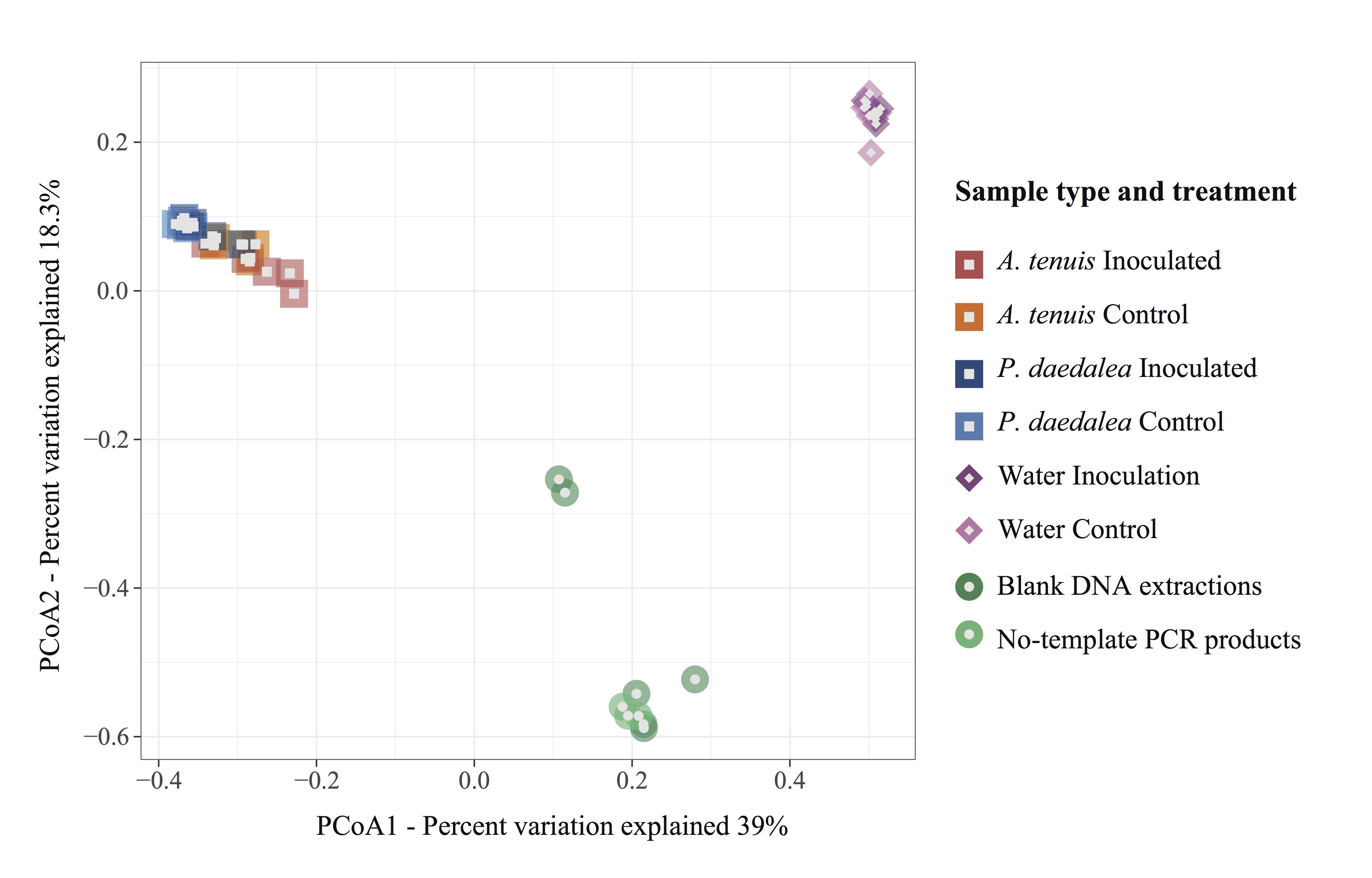


**Supplementary Figure 5.** Principal Coordinate Analysis **(**PCoA) visualization of bacterial communities associated with coral recruits, water samples, blank extractions and no-template PCR controls. Coral recruits, water samples and negative controls (blank extractions, no-template PCRs) cluster away from each other.

| **Supplementary Table 2** Identifiers and average relative abundance (Av. rel. ab.) of the most prevalent bacterial ASVs in the negative control samples (6 blank DNA extractions and 3 no-template PCR products). | | | | |
| --- | --- | --- | --- | --- |
| **ASV identifiers** | **Family** | **Genus** | **Av. rel. ab. Blanks** | **Av. rel. ab. No-template PCRs** |
|  |  |  |  |  |
| 7fc531142a541cd59c8c3bf53006bc12 | Burkholderiaceae | Burkholderia-paraburkholderia | 41 % | 1.2 % |
|  |  |  |  |  |
| 79fea7073a32159fbf592c02ee449582 | Burkholderiaceae | Ralstonia | 27.9 % | 62.9 % |
|  |  |  |  |  |
| 02c81cd7bf5d96002f579f3e947a6d68 | Burkholderiaceae | Burkholderia-paraburkholderia | 2.4 % | 0 % |
|  |  |  |  |  |
| c029262913246bc9941aee3989307e4a | Oxalobacteraceae | Unidentified | 1.9 % | 9.3 % |

**Supplementary Figure 6.** Bacterial community composition in the seven inocula administered to the coral recruits. In each inoculum, the seven strains were present but with different relative abundances. Left: relative abundance of bacterial cultures on the basis of optical density at 660 nm determined by NanoDrop; right: relative abundance of reads per sample obtained after metabarcoding of the 16S rRNA genes.

**Supplementary Figure 7.** Relative abundance of the 15 most prevalent bacterial classes in recruits and water samples across all treatments.

**
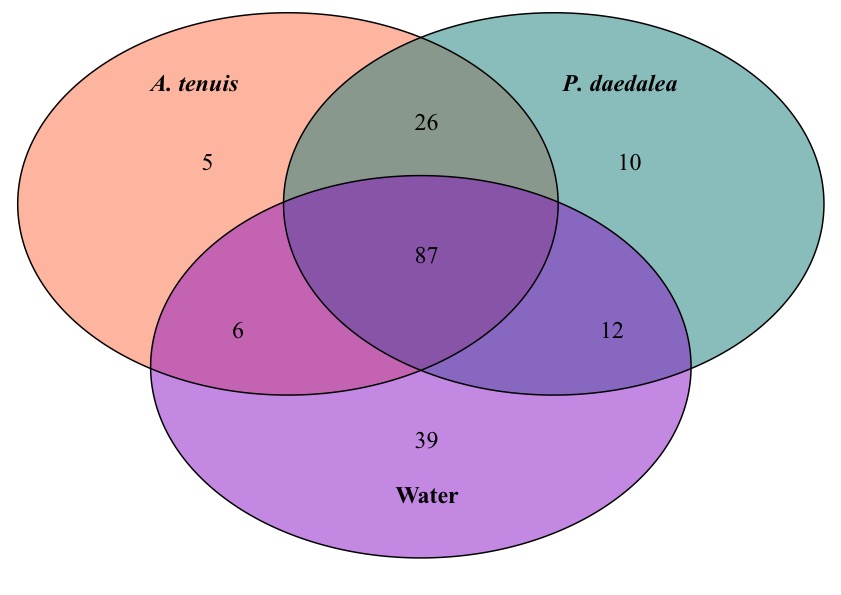
**

**Supplementary Figure 8.** Number of bacterial families uniquely present or shared among coral and water samples.
